# Yo-Yo Reflux Into a Blind-Ending Branch of a Bifid Ureter: A Case Report

**DOI:** 10.7759/cureus.46970

**Published:** 2023-10-13

**Authors:** Peter Estaphanous, Francis X Keeley

**Affiliations:** 1 Urology, North Bristol NHS Trust, Bristol, GBR

**Keywords:** urological anomaly, congenital abnormalities, duplicated ureter, incomplete double ureter, duplex systems

## Abstract

A bifid ureter is an uncommon congenital anomaly. It develops through abnormal branching of the ureteric bud in utero and represents incomplete duplication of the collecting system. However, a bifid ureter with a blind-ending branch is a rare variant.

We present the case of a 26-year-old female who presented with recurrent urinary tract infections and an episode of pyelonephritis. Radiological imaging revealed a blind-ending branch of a bifid ureter with Yo-Yo reflux.

This report demonstrates laparoscopic evidence of the reflux and management of this rare congenital anomaly.

## Introduction

A bifid ureter is a congenital abnormality characterized by a Y-shaped bifurcation in the urinary collecting system [1]. If one branch of the Y-shaped bifurcation fails to reach the metanephric mesenchyme, this results in a blind-ending branch of the bifid ureter, which is a very rare anomaly [2].

Most of the cases are asymptomatic and discovered incidentally; therefore, the exact incidence cannot be determined. However, some cases present with recurrent urinary tract infections (UTIs) and nonspecific abdominal pain [3]. The reflux of the urine from one branch to the blind-ending branch, rather than drainage of the urine downward to the urinary bladder, can cause these symptoms. This reflux is described as Yo-Yo reflux [4]. Management of symptomatic cases includes conservative and surgical approaches [5].

We present a case with a blind-ending branch of a bifid ureter requiring laparoscopic removal. Yo-Yo reflux was demonstrated in this case.

## Case presentation

A 26-year-old female who was diagnosed with bilateral duplex systems as a child was referred for recurrent UTIs and a recent episode of right-sided pyelonephritis. She reported symptoms of dysuria, frequency, severe fatigue, and right-sided flank pain.

She underwent several investigations, including a computed tomography (CT) urogram and a right retrograde pyelogram.

**Figure 1:**
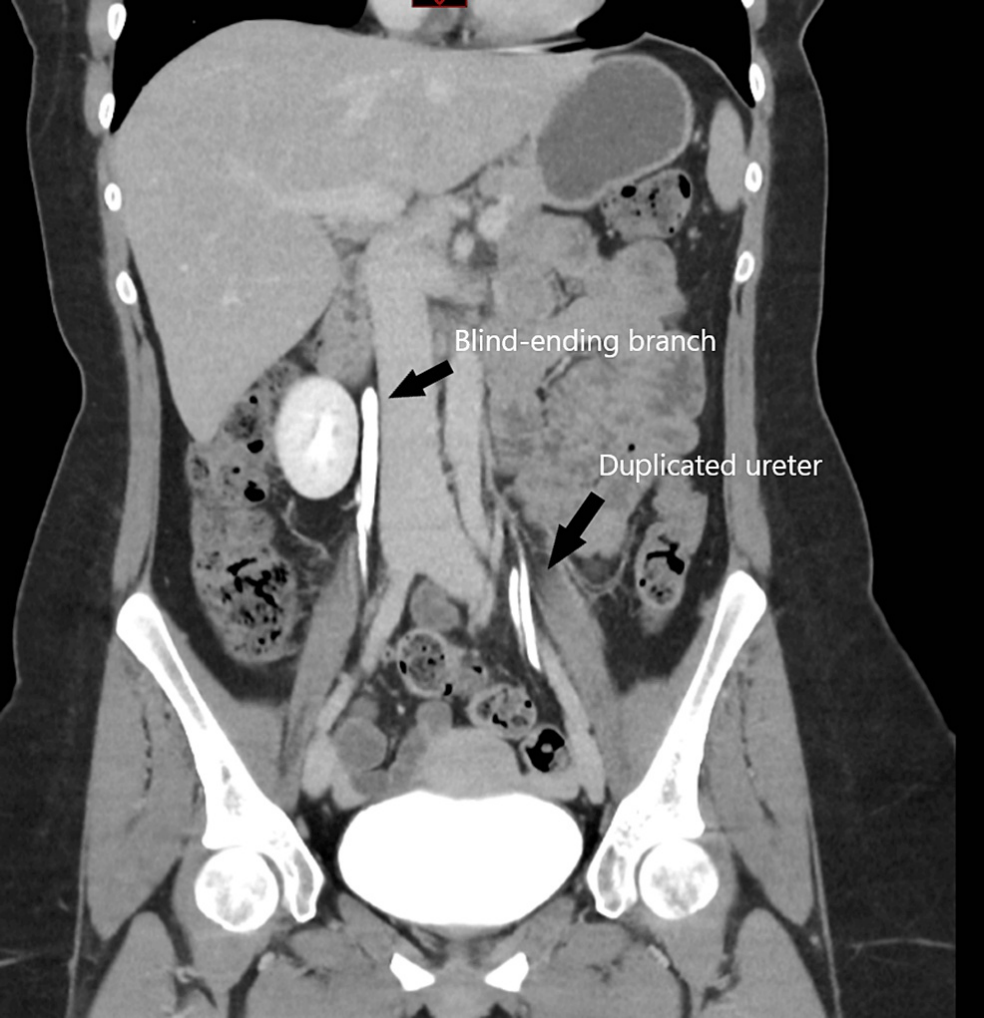
Computed tomography (CT) scan of the urinary tract with contrast showing the blind-ending branch of the bifid ureter on the right side and a duplicated ureter on the left side (sagittal plane).

The CT urogram revealed bilateral duplex kidneys, with the left ureters appearing to join together at the level of the iliac vessels. However, the right ureter showed a blind-ending branch that went up to the level of the kidney but did not fuse with the renal parenchyma (Figure 1). The presence of contrast at the top end of the blind-ending branch suggested reflux of urine from the bladder or the ureter to the blind-ending branch. This reflux is described in the literature as Yo-Yo reflux.

**Figure 2:**
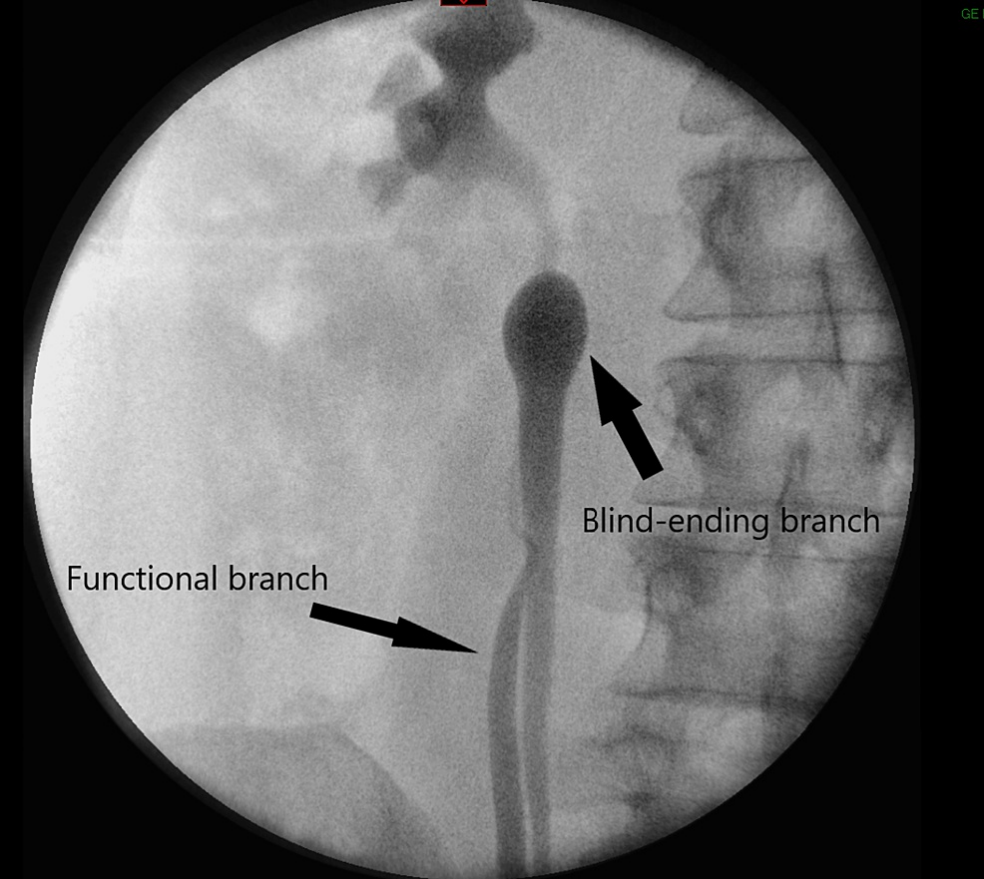
Retrograde study showing the blind-ending branch and the functional branch of the right bifid ureter.

**Figure 3:**
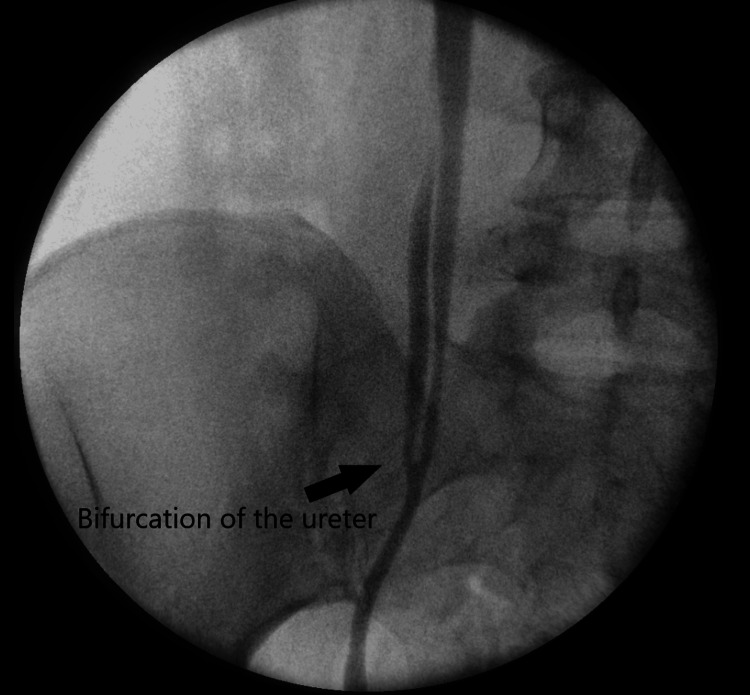
Retrograde study showing the bifurcation of the right ureter into the blind-ending branch and the functional branch.

The retrograde pyelogram confirmed duplication of the right ureter at the level of iliac vessels with a medial blind ending branch (Figures 2-3). Reflux of the contrast to the blind-ending branch was clearly seen. Vesicoureteric reflux was ruled out by micturating cystourethrogram.

Management options were discussed with the patient, including conservative approach and surgical removal of the blind-ending branch. As the patient was complaining of recurrent symptoms, she preferred the surgical approach. Laparoscopic ureterectomy of the blind-ending branch was offered and explained in detail. Risks of the operation was explained, including the risk of having no benefit as the occurrence of recurrent UTI could be linked to other reasons. The patient agreed to proceed with the operation.

During the operation, the right duplex ureters were identified and followed down to the Y junction at the level of iliac vessels. Interestingly, Yo-Yo reflux was detected by noticing the peristalsis moving from the normal ureter to the medial blind-ending ureter. The blind-ending ureter was excised at the level of bifurcation, leaving the upper normal ureter in continuity with the distal ureter.

Postoperatively, the patient recovered well. On subsequent follow-up, the patient reported improvement of her persistent loin pain and fatigue with no recurrence of UTIs.

## Discussion

The case highlights the rare anomaly of a blind-ending branch of a bifid ureter with observed evidence of Yo-Yo reflux. A bifid ureter is a relatively uncommon condition resulting from abnormal branching of the ureteric bud during embryonal development. In most cases, both branches of the bifurcation join the metanephric mesenchyme and fuse with the renal pelvis, forming a Y-shaped structure. However, when one branch fails to reach the metanephric mesenchyme, a blind-ending branch is formed [6]. The first reported case of this anomaly was described by Herbert in 1904 [7].

The clinical presentation varies. Some patients remain asymptomatic, others may experience recurrent UTIs, pyelonephritis, and flank or abdominal pain [8]. The reflux of the urine from the functional ureter to the blind-ending branch could be a contributor for the symptoms [9].

Yo-Yo reflux has been described in literature to occur as a result of abnormal peristalsis propagation from the functional branch to the blind-ending branch or due to the difference in the pressure gradient between the two branches [10].

It is quite challenging to demonstrate Yo-Yo reflux radiologically. In our case, however, the radiological imaging has demonstrated the evidence of the reflux. The CT urogram showed the presence of the contrast in the blind-ending branch, and the retrograde pyelogram demonstrated reflux of the contrast from the lateral functional branch to the medial blind-ending branch.

The presence of Yo-Yo reflux was not only confirmed through radiological imaging but also directly observed and recorded during the laparoscopic procedure. The peristaltic movement of urine from the functional branch to the blind-ending branch was visually identified (Video 1).

**Video 1:** Yo-Yo reflux. Yo-Yo reflux was observed intraoperatively; the propagation of peristalsis with dilatation of the medial blind-ending branch following emptying of the lateral functional branch is clearly evident.

Laparoscopic ureterectomy of the blind-ending branch was performed successfully (Video 2). During the operation, the blind-ending branch was identified and excised at the level of the bifurcation, leaving the functional branch to remain in continuity with the distal ureter. Resolution of the symptoms following the operation confirms the relation between the condition and the symptoms.

**Video 2:** Laparoscopic uretrectomy of a blind-ending branch of a bifid ureter.

## Conclusions

A bifid ureter with a blind-ending branch is a rare variant of ureteric duplication. The demonstration of Yo-Yo reflux into the blind-ending branch is unusual. It can be challenging to prove the reflux. Our case demonstrates a radiological and a visual evidence of the reflux during laparoscopy.

Asymptomatic cases may not need treatment; however, surgical intervention might be required in symptomatic cases. Our case provides evidence of the effectiveness of laparoscopic ureterectomy as a management option for this rare anomaly.
